# Comparative predictive performance of anticholinergic burden scales for anticholinergic-related hospitalisation and emergency department visits in older adults: a population-based study of model development and temporal validation

**DOI:** 10.1007/s11096-026-02145-9

**Published:** 2026-04-17

**Authors:** Valentina M. Srikartika, David Youens, Rachael Moorin, Ninh Ha

**Affiliations:** 1 Health Economics and Data Analytics, School of Population Health, Curtin University, Perth, WA 6102 Australia; 2 Pharmacy Program Study, Faculty of Mathematics and Natural Science, Lambung Mangkurat University, Banjarbaru, South Kalimantan 70714 Indonesia; 3 School of Population and Global Health, The University of Western Australia, Crawley, WA 6001 Australia

**Keywords:** Anticholinergic burden scales, Calibration, Discrimination, Predictive performance, Risk prediction, Validation

## Abstract

**Introduction:**

Anticholinergic burden scales are widely used to guide medication review in older adults, yet their ability to predict clinically important adverse outcomes remains uncertain, as most evidence is based on associations rather than validated risk prediction. In the absence of a gold standard, the clinical value of these scales depends on whether they can discriminate between individuals at risk and provide accurate absolute risk estimates.

**Aim:**

To compare the predictive performance of six anticholinergic burden scales for hospitalisation/emergency department (ED) visits related to anticholinergic adverse effects, assess internal and temporal validation of cognitive impairment models across scales, and examine the risk gradient across increasing anticholinergic burden levels for cognitive impairment across scales.

**Method:**

A retrospective cohort study using linked population-level administrative health data of adults aged ≥ 65 years in Western Australia (2015–2019). Development cohorts were from 2015–16 (N = 323,682) and 2016–17 (N = 334,304), and the temporal validation cohort was from 2017–18 (N = 330,684). Six anticholinergic burden scales were calculated annually. Logistic regression models with common predictor structure including demographic and clinical predictors were used to predict any hospitalisation/ED visit related to anticholinergic adverse events (falls/fractures/dizziness, cognitive impairment, or constipation/urinary retention). Model performance was assessed using the c-statistic, calibration slope, and Brier score. Cognitive impairment models were further evaluated using bootstrap internal validation (200 replications), temporal validation, and predicted risk estimation across exposure deciles.

**Results:**

Across initial model comparisons, Korean Anticholinergic Burden Scale (KABS) showed the highest c-statistics for each outcome, although between-scale differences were small. Cognitive impairment showed the highest discrimination across scales and was selected for further validation. In temporal validation, c-statistics for cognitive impairment ranged from 0.795 to 0.806, with the highest value observed for KABS, and calibration slopes ranging from 1.024 to 1.032 across scales. Predicted risk of cognitive impairment increased across exposure deciles for all scales, with the highest-decile risk ranging from 5.27% for TSDD-SAMS to 7.58% for KABS.

**Conclusion:**

KABS showed slightly higher and more consistent predictive performance than the other scales, particularly for cognitive impairment, although between-scale differences were modest. Presenting validated absolute risk estimates across exposure groups may improve clinical interpretability and support risk stratification, but wider validation and clinical judgement remain essential before routine use.

**Supplementary Information:**

The online version contains supplementary material available at 10.1007/s11096-026-02145-9.

## Impact statements

- The predictive performance of anticholinergic burden scales varied across adverse outcomes, indicating that different scales may identify risk differently in older adults.
- KABS showed slightly higher and more consistent predictive performance than the other scales, although absolute differences were modest.
- Validated absolute risk estimates across anticholinergic burden levels for cognitive impairment may improve clinical interpretability and support risk stratification to inform medication review and deprescribing.

## Introduction

Anticholinergic medications block the action of acetylcholine in central and peripheral tissues and are used to treat conditions such as Parkinson’s disease, depression, allergy, overactive bladder, and some psychiatric disorders [1]. These medications are often prescribed to older adults, despite their well-documented risks. Population-based studies have reported that between 32.8 and 64.3% of individuals aged ≥ 65 years are dispensed at least one anticholinergic medication annually, with approximately 12.1% exposed to clinically significant levels [2–5]. Such exposure is consistently linked with adverse outcomes, including cognitive impairment [6], falls and fractures [7, 8], and an increased risk of hospitalisation and mortality [9, 10].

Anticholinergic burden refers to the cumulative effect of taking one or more anticholinergic medications [6]. To quantify this burden and guide safer prescriptions or medication reviews, at least 28 scales have been developed [11]. These tools vary in terms of medications included, potency definition (via expert consensus or pharmacological properties), and burden calculation (incorporating dose, potency, duration, or using average vs. aggregate scores) [11]. This methodological diversity reflects the complexity of anticholinergic exposure but also limits direct comparability and the ability to draw firm conclusions about which scale performs best in clinical practice. For example, the Anticholinergic Cognitive Burden (ACB) scale has been linked to stronger dose–response relationships with cognitive outcomes, Emergency Department (ED) visits, and hospitalisation, whereas the Drug Burden Index (DBI) better predicts fractures [12, 13], and the Korean Anticholinergic Burden Scale (KABS) has demonstrated stronger associations with urinary retention [14]. These findings suggest that anticholinergic burden is a multidimensional construct, with different scales capturing distinct pharmacological and clinical domains.

Although anticholinergic burden has been consistently associated with adverse outcomes, most studies have focused on measures of association (e.g. odds ratios) rather than whether burden scales can provide accurate individual risk estimates. Key aspects of predictive performance, especially discrimination (i.e., a scale's ability to distinguish between those at risk) and calibration (i.e., how closely predicted risks match observed outcomes) are often absent from the literature [15–17]. Even among studies that assess predictive performance of anticholinergic burden scales, internal and external validations remain uncommon [18–22], and when calibration is examined, findings often indicate over-prediction during external validation [23]. In the absence of a universal gold standard, comparative assessment of predictive performance across anticholinergic burden scales is needed to determine which scales may better predict anticholinergic adverse outcomes and provide clinically reliable risk estimates across populations.

To our current knowledge, absolute predicted probabilities across anticholinergic burden exposure levels have not been reported in this area. Such estimates may be more clinically interpretable than odds ratios alone and may better support risk stratification and prioritisation of older adults for medication review and deprescribing [24].

### Aim

To compare the predictive performance of six anticholinergic burden scales for hospitalisation/emergency department (ED) visits related to anticholinergic adverse effects, assess internal and temporal validation of cognitive impairment models across scales, and examine the risk gradient across increasing anticholinergic burden levels for cognitive impairment across scales.

## Method

### Study design and data source

This study used whole-of-population-linked administrative health data to construct a retrospective longitudinal cohort of individuals in Western Australia (WA) between July 1, 2015, and June 30, 2019. Individual-level data were linked by the Curtin Centre for Data Linkage [25], combining the WA Data Linkage System datasets [26] with Commonwealth data from the Australian Institute of Health and Welfare. Six routinely collected datasets were used: (1) the Medicare Enrolments File for residency and cohort eligibility; (2) Medicare Benefits Schedule (MBS) for demographics and GP service claims; (3) Pharmaceutical Benefits Scheme (PBS) for subsidised prescription records; (4) Hospital Morbidity Data Collection (HMDC) for hospital records: (5) Emergency Department Data Collection (EDDC) for ED presentations; and (6) National Death Index (NDI) for mortality. Data were accessed in a Secure Unified Research Environment (SURE).

### Study population

Annual panel data from 2015–16 to 2017–18 were constructed from Medicare-registered individuals aged 65–100 years residing in WA during the study period with at least one year of follow-up. Patients without any MBS, PBS, or HMDC/EDDC records in the relevant year were excluded. Three annual cohorts were constructed: 2015–16 and 2016–17 for model development (N = 323,682 and 334,304, respectively) and 2017–18 for temporal validation (N = 330,684). A couplet design, in which each cohort comprised an exposure year followed by an outcome year, was applied consistently across all cohorts. Details of annual cohort construction and the panel couplet structure are presented in Supplementary file 1.

### Anticholinergic burden exposure

Six scales for measuring anticholinergic exposure were selected based on prior scoring evaluations and feasibility for PBS application [11]: the modified Drug Burden Index (mDBI) [27], the modified Anticholinergic Cognitive Burden (mACB) [28], the modified Anticholinergic Risk Scale (mARS) [29], Coupland List [30], the Total Standardised Daily Dose–Summated Anticholinergic Medications Scale (TSDD-SAMS) [31], and the Korean Anticholinergic Burden Scale (KABS) [32]. The scales were calculated annually using ATC codes and the original scoring rules (Supplementary File 2). Scores were reported as average daily burden (mDBI, mACB, mARS, KABS) or cumulative standardised doses (TSDD-SAMS, Coupland List). Scores were categorised into scale-specific deciles within cohorts. These deciles served as distribution-based exposure groups to support comparability across scales with different scoring ranges. No exposure was retained as reference category, and positive exposure was grouped into increasing deciles of anticholinergic burden.

### Outcomes

Participants were followed for 12 months after the exposure assessment to identify hospitalisations/ED visits related to anticholinergic adverse events, identified using ICD-10-AM codes recorded in any diagnostic field from the HMDC and EDDC (Supplementary File 3) based on clinical relevance and prior literature [14, 33–36]. The primary outcome was a composite of (1) falls, fractures, or dizziness, (2) cognitive impairment, and (3) constipation or urinary retention. This composite was used to summarise clinically meaningful acute presentations plausibly related to anticholinergic effects. Each component was also analysed separately. Hospitalisation and ED visits were assessed together to capture acute care presentations across inpatient and emergency care, with ED records resulting in admission or transfer excluded to avoid double counting. Fall-related hospitalisations were identified using external cause codes, and fractures were included only if they co-occurred with falls.

### Predictors

Predictors were constructed at baseline year for each cohort, and selected based on temporal precedence, definitional clarity [37], and completeness [38]. Sociodemographic factors included age group (65–74, 75–84, 85–94, and ≥ 95 years), sex, socioeconomic status (Index of Relative Socio-Economic Disadvantage quintiles from the Census-based Socio-Economic Indexes for Areas (SEIFA) [39]), and service accessibility (Accessibility/Remoteness Index of Australia (ARIA) [40]). Multimorbidity was measured using the Multipurpose Australian Comorbidity Scoring System (MACSS) [41]. All predictors were categorical.

## Statistical analysis

Population characteristics were summarised by outcome status, and categorical variables were compared using the chi-squared test.

### Model development

Model development was conducted in two sequential steps using the 2015–16 and 2016–17 cohorts.

Step 1: Development of the base model.

A common base model was established for each outcome without inclusion of any anticholinergic burden scale. Candidate predictors and clinically plausible interaction terms between covariates were evaluated using penalised logistic regression with the least absolute shrinkage and selection operator (LASSO). Candidate shrinkage parameters were assessed, and λ = 0.001 was selected based on model stability. Predictors and interaction terms with non-zero coefficients were retained and subsequently refitted using multivariable logistic regression to define the final base model across outcomes (Supplementary file 4).

Step 2: Evaluation of anticholinergic burden scales.

Each anticholinergic burden scale was then added separately to the base model to construct scale-specific models for each outcome. The same base model structure was used for all scale-specific models to ensure comparability across scales. Apparent model performance (i.e. performance in the same data used for model development)[15] was assessed in terms of discrimination, calibration, and overall predictive performance using the c-statistic, calibration slope, and Brier score, respectively. Model fit was compared using the Akaike Information Criterion (AIC), and the difference in AIC (ΔAIC) was calculated by comparing the base model with the corresponding scale-specific model. Based on these comparisons, cognitive impairment was selected as the outcome for further validation across all scales.

Step 3: Internal Validation.

Internal validation was conducted for the cognitive impairment models using bootstrap resampling for each scale (200 replications). In each bootstrap replicate, the final selected logistic model was refitted in the bootstrap sample and evaluated in both the bootstrap sample and the original development dataset to estimate optimism. Mean optimism was calculated as the difference between bootstrap-sample and test-sample performance and subtracted from the original model performance to obtain optimism-adjusted estimates of the c-statistic, calibration slope, and Brier score [37, 42].

Step 4: Temporal Validation.

Temporal validation was performed using the 2017–18 cohort. Regression coefficients from the development models were applied to the validation dataset to generate predicted probabilities for each scale-specific cognitive impairment model. Temporal validation performance was assessed using the c-statistic, discrimination slope, calibration-in-the-large, calibration slope, Brier score, scaled Brier score, and expected-to-observed ratio. Calibration plots were constructed by plotting observed and predicted risks across deciles of predicted probability, together with a smoothed (lowess) calibration curve and the ideal reference line.[15, 38].

### Predicted risk estimation

Predicted risks for cognitive impairment were calculated in the validation dataset for each scale using the development-model coefficients. Mean predicted probabilities and corresponding 95% confidence intervals were summarised within each exposure decile to illustrate risk gradients across increasing burden levels.

**Fig. 1:**
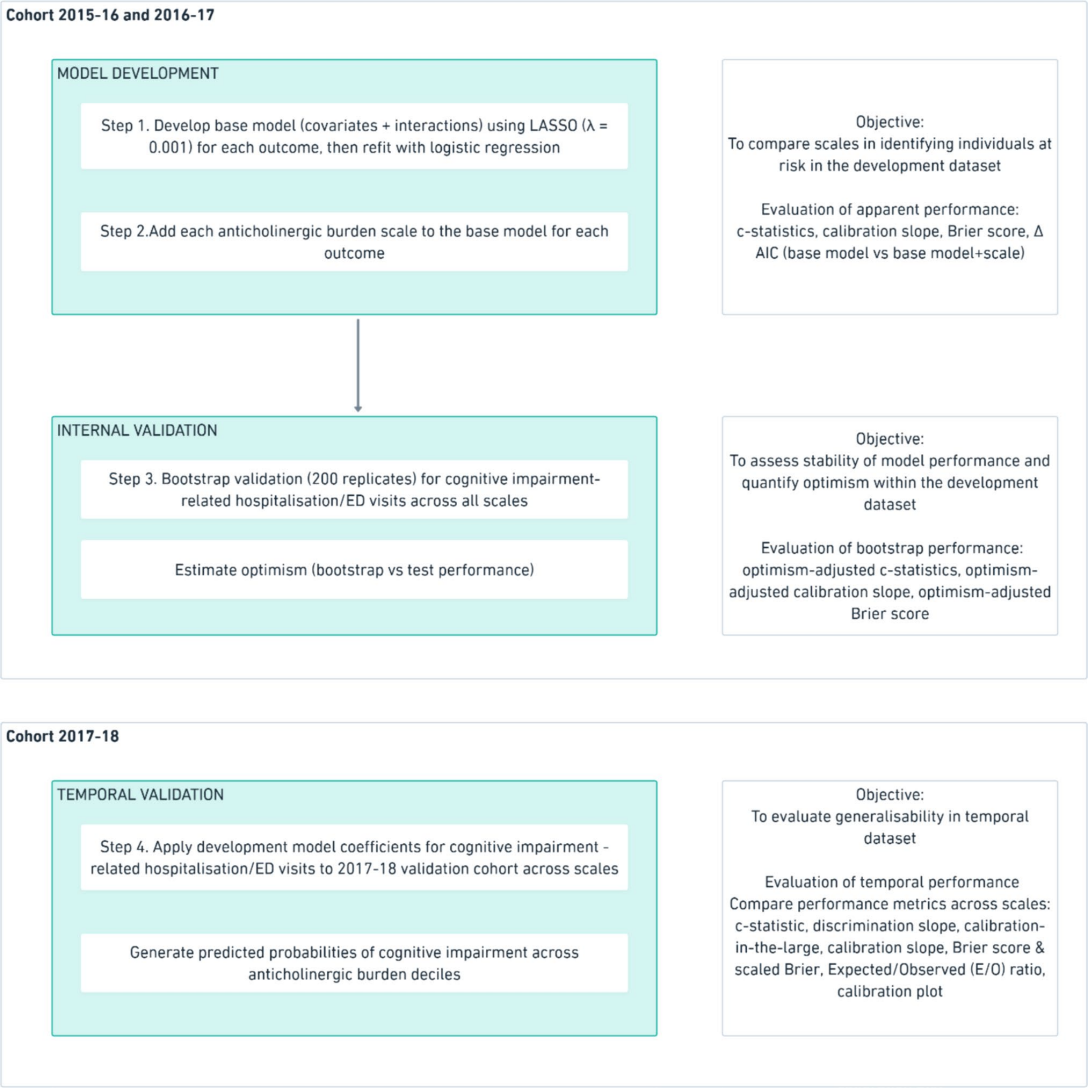
The overall modelling and validation workflow

The overall modelling and validation workflow is illustrated in Fig. 1.

All analyses were conducted using Stata version 18 [43].

### Ethics approval

This study was approved by the Australian Institute of Health and Welfare, the Western Australia Department of Health, and Curtin University Human Research Ethics Committees (Approval number: HRE2024-0695).

## Results

### Participant characteristics

Table 1 shows baseline characteristics of development and validation cohorts by primary outcome (hospitalisation/ED visit related to anticholinergic adverse events). Baseline characteristics for the other outcomes are presented in Supplementary File 5. Individuals with the primary outcome were older, more often female, socioeconomically disadvantaged, and had higher comorbidity burden across cohorts. Among people dispensed with anticholinergic medications, the proportion experiencing the primary outcome ranged from 13.4 to 15.6% in 2015–16, 13.7% to 16.2% in 2016–17, and 13.9% to 16.3% in 2017–18. The lowest event proportions were observed for mACB, whereas the highest were for mARS. All group comparisons were statistically significant.

**Table 1:** Baseline characteristics of development and validation datasets based on outcome of any hospitalisation/ED visit related to anticholinergic adverse events

Variables	Categories/Unit	Development dataset 2015–16 (n = 323,682)					Development dataset 2016–17 (n = 334,304)					Validation dataset 2017–18 (n = 330,684)				
		No Hospitalisation /ED visit (n,%)		Yes Hospitalisation /ED visit (n,%)		*p* value	No Hospitalisation /ED visit (n,%)		Yes Hospitalisation /ED visit (n,%)		*p* value	No Hospitalisation /ED visit (n,%)		Yes Hospitalisation /ED visit (n,%)		*p* value
Sex	Female	153,988	90.0	17,145	10.0	< 0.001	158,418	89.8	18,036	10.2	< 0.001	156,952	89.4	18,589	10.6	0.015
	Male	138,080	90.5	14,469	9.5		142,716	90.4	15,134	9.6		139,116	89.7	16,027	10.3	
Age	65–74	179,274	94.5	20,579	5.6	< 0.001	185,238	94.4	10,937	5.6	< 0.001	178,970	94.0	11,383	6.0	< 0.001
	75–84	84,956	87.5	24,240	12.5		87,671	87.3	12,729	12.7		88,400	86.9	13,337	13.1	
	85–94	26,236	76.0	15,445	24.0		26,568	75.4	8,686	24.6		26,923	74.8	9,049	25.2	
	95 +	1,602	70.2	1,250	29.8		1,657	67.0	818	33.1		1,775	67.7	847	32.3	
SEIFA*	Least disadvantage	84,547	90.3	9,068	9.7	< 0.001	87,469	90.2	9,465	9.8	< 0.001	86,090	90.7	9,646	10.1	< 0.001
	Less disadvantage	44,855	89.6	5,194	10.4		46,064	89.3	5,537	10.7		45,478	89.6	5,723	11.2	
	Moderate disadvantage	52,460	90.3	5,649	9.7		54,275	90.3	5,840	9.7		53,444	90.3	6,214	10.4	
	High disadvantage	78,122	90.7	8,030	9.3		80,457	90.5	8,477	9.5		79,106	90.5	8,932	10.2	
	Highest disadvantage	31,846	89.7	3,657	10.3		32,595	89.5	3,846	10.6		31,702	89.5	4,079	11.4	
ARIA*	Major cities	215,459	89.7	24,829	10.3	< 0.001	221,656	89.5	25,933	10.5	< 0.001	218,279	89.0	26,906	11.0	< 0.001
	Inner regional	41,538	92.2	3,507	7.8		43,018	91.8	3,862	8.2		42,599	91.5	3,983	8.6	
	Outer regional	25,713	91.4	2,425	8.6		26,695	91.6	2,444	8.4		26,016	90.5	2,735	9.5	
	Remote	5,421	91.9	475	8.1		5,578	91.2	536	8.8		5,476	90.9	551	9.1	
	Very Remote	3,928	91.3	376	8.7		4,176	91.4	395	8.6		3,691	89.4	439	10.6	
MACCS	0	69,992	94.7	3,894	5.3	< 0.001	70,857	95.1	3,664	4.9	< 0.001	64,356	94.7	3,615	5.3	< 0.001
	1–4	131,050	92.9	10,550	7.2		140,666	92.8	10,860	7.2		137,622	92.5	11,146	7.5	
	5–9	72,321	85.7	12,071	14.3		75,972	85.5	12,868	14.5		79,377	85.7	13,234	14.3	
	10 +	12,705	71.4	5,099	28.6		13,639	70.2	5,778	29.8		14,713	69.0	6,621	31.0	
Anticholinergic	TSDD-SAMS	35,691	85.7	5,976	14.3	< 0.001	36,509	85.3	6,290	14.7	< 0.001	36,847	84.8	6,584	15.2	< 0.001
medication	mDBI	39,066	85.4	6,680	14.6		40,012	85.0	7,038	15.0		40,532	84.5	7,434	15.5	
dispensing	Coupland List	39,143	85.3	6,746	14.7		39,989	84.9	7,107	15.1		40,555	84.6	7,397	15.4	
	mARS	62,736	84.4	11,615	15.6		63,000	83.8	12,156	16.2		64,206	83.7	12,521	16.3	
	mACB	145,997	86.6	22,575	13.4		149,811	86.4	23,683	13.7		152,635	86.1	24,586	13.9	
	KABS	122,165	85.7	20,402	14.3		125,801	85.3	21,667	14.7		128,139	85.1	22,431	14.9	

*P*-values were calculated using the chi-square test to assess the association between each baseline characteristic and the occurrence of any anticholinergic-related hospitalisation or ED visit. * Small numbers without SEIFA/ARIA where the recorded residential postcode did not map to SEIFA / ARIA files. SEIFA, Socio-Economic Indexes for Areas; ARIA, Accessibility/Remoteness Index of Australia; MACSS, Multipurpose Australian Comorbidity Scoring System; TSDD-SAMS, Total Standardised Daily Dose–Summated Anticholinergic Medications Scale; mDBI, modified Drug Burden Index; mARS, modified Anticholinergic Risk Scale; mACB, modified Anticholinergic Cognitive Burden (mACB); KABS, Korean Anticholinergic Burden Scale

### Comparative model performance

Table 2 summarises performance across six anticholinergic burden scales. In model comparisons, KABS showed the highest c-statistics for each outcome, though differences between scales were modest. For hospitalisation/ED visits related to anticholinergic adverse effects, c-statistics ranged from 0.726 to 0.737. For cognitive impairment, discrimination was highest, with c-statistics from 0.791 to 0.804. Calibration slopes and Brier scores were similar across scales within each outcome. All scale-specific models showed lower AIC values than base models, indicating improved fit after including anticholinergic burden, with greatest reductions seen for KABS across outcomes. Based on these comparisons, cognitive impairment was selected for further validation across all scales.

**Table 2:** Apparent predictive performance across anticholinergic burden scales for hospitalisation and ED outcomes in the development cohorts

Anticholinergic burden scales	Measures	Any Hospitalisations and ED Visits related anticholinergic adverse effects	Fall, fractures, and dizziness	Cognitive impairment	Constipation and urinary retention
TSDD-SAMS	c-statistics	0.726	0.733	0.791	0.724
	Calibration Slope	1.000	1.000	1.000	1.000
	Brier Score	0.082	0.051	0.023	0.044
	ΔAIC	-868.9	-367.8	-310.5	-599.5
Coupland List	c-statistics	0.727	0.733	0.792	0.725
	Calibration Slope	1.000	1.000	1.000	0.999
	Brier Score	0.082	0.051	0.023	0.044
	ΔAIC	-1157.1	-525.8	-453.0	-710.1
Modified DBI	c-statistics	0.727	0.733	0.792	0.725
	Calibration Slope	1.000	1.000	1.000	1.000
	Brier Score	0.082	0.051	0.023	0.044
	ΔAIC	-1091.5	-508.4	-388.1	-658.6
KABS	c-statistics	0.737	0.741	0.804	0.733
	Calibration Slope	1.000	1.000	1.000	0.999
	Brier Score	0.082	0.051	0.023	0.044
	ΔAIC	-4552.5	-2169.1	-2111.1	-2158.1
Modified ACB	c-statistics	0.734	0.740	0.801	0.729
	Calibration Slope	1.000	1.000	1.000	1.000
	Brier Score	0.082	0.051	0.023	0.044
	ΔAIC	-3628.0	-1865.6	-1738.6	-1533.1
Modified ARS	c-statistics	0.730	0.736	0.796	0.728
	Calibration Slope	1.000	1.000	1.000	1.000
	Brier Score	0.082	0.051	0.023	0.044
	ΔAIC	-2077.8	-928.6	-983.1	-1132.8

TSDD-SAMS, Total Standardised Daily Dose–Summated Anticholinergic Medications Scale; mDBI, modified Drug Burden Index; mARS, modified Anticholinergic Risk Scale; mACB, modified Anticholinergic Cognitive Burden (mACB); KABS, Korean Anticholinergic Burden Scale; AIC, The Akaike Information Criterion

### Internal and temporal validation of cognitive impairment models across scales

Table 3 presents validation performance of cognitive impairment models across scales. After bootstrapping internal validation (200 replications), optimism-adjusted c-statistics ranged from 0.790 for TSDD-SAMS to 0.803 for KABS. Optimism-adjusted calibration slopes were 0.997 to 0.998, and Brier scores remained 0.023 across models, indicating minimal optimism.[15].

**Table 3:** Internal and temporal validation performance of anticholinergic burden scales for predicting cognitive impairment-related hospitalisation/ED visits

Performance measure		Internal validation, estimate (95% CI)	Temporal validation
*TSDD-SAMS*			
Overall	Brier score	0.023 (0.023–0.024)	0.025
	Brier scaled		4.148
Discrimination	C stat	0.790 (0.786–0.793)	0.795
	Discrimination slope		0.039
Calibration	Calibration-in-the-large		-0.001
	Calibration slope	0.997 (0.982–1.012)	1.032
	Average predicted risk (%)		2.597
	Observed risk (%)		2.697
	Over-estimation incidence (%)		0.100
*Coupland List*			
Overall	Brier score	0.023 (0.023–0.024)	0.025
	Brier scaled		4.164
Discrimination	C stat	0.792 (0.788–0.795)	0.796
	Discrimination slope		0.040
Calibration	Calibration-in-the-large		-0.001
	Calibration slope	0.998 (0.982–1.011)	1.031
	Average predicted risk (%)		2.600
	Observed risk (%)		2.697
	Over-estimation incidence (%)		0.097
*mDBI*			
Overall	Brier score	0.023 (0.023–0.024)	0.025
	Brier scaled		4.130
Discrimination	C stat	0.791 (0.788–0.795)	0.795
	Discrimination slope		0.039
Calibration	Calibration-in-the-large		-0.001
	Calibration slope	0.997 (0.983–1.016)	1.030
	Average predicted risk (%)		2.597
	Observed risk (%)		2.697
	Over-estimation incidence (%)		0.100
*KABS*			
Overall	Brier score	0.023 (0.023–0.024)	0.025
	Brier scaled		4.453
Discrimination	C stat	0.803 (0.801–0.807)	0.806
	Discrimination slope		0.043
Calibration	Calibration-in-the-large		-0.001
	Calibration slope	0.997 (0.982–1.012)	1.024
	Average predicted risk (%)		2.590
	Observed risk (%)		2.697
	Over-estimation incidence (%)		0.107
*mARS*			
Overall	Brier score	0.023 (0.023–0.024)	0.025
	Brier scaled		4.212
Discrimination	C stat	0.796 (0.792–0.799)	0.799
	Discrimination slope		0.040
Calibration	Calibration-in-the-large		-0.001
	Calibration slope	0.997 (0.981–1.011)	1.030
	Average predicted risk (%)		2.581
	Observed risk (%)		2.697
	Over-estimation incidence (%)		0.116
*mACB*			
Overall	Brier score	0.023 (0.023–0.024)	0.025
	Brier scaled		4.384
Discrimination	C stat	0.801 (0.798–0.804)	0.804
	Discrimination slope		0.042
Calibration	Calibration-in-the-large		-0.001
	Calibration slope	0.997 (0.984–1.013)	1.027
	Average predicted risk (%)		2.593
	Observed risk (%)		2.697
	Over-estimation incidence (%)		0.104

Internal validation values are optimism-adjusted estimates from bootstrap resampling (200 replications); values in parentheses are 95% confidence intervals. Temporal validation was assessed in the 2017–18 cohort

In temporal validation, c-statistics for cognitive impairment ranged from 0.795 to 0.806 across scales, with the highest for KABS; calibration slopes ranged from 1.024 to 1.032. Average predicted risks were 2.581% to 2.600%, compared with an observed risk of 2.697%, and overestimation incidence ranged from 0.097% to 0.116%. These findings indicate acceptable temporal stability across models, with only small between-scale differences.

### Calibration plots

**Fig. 2:**
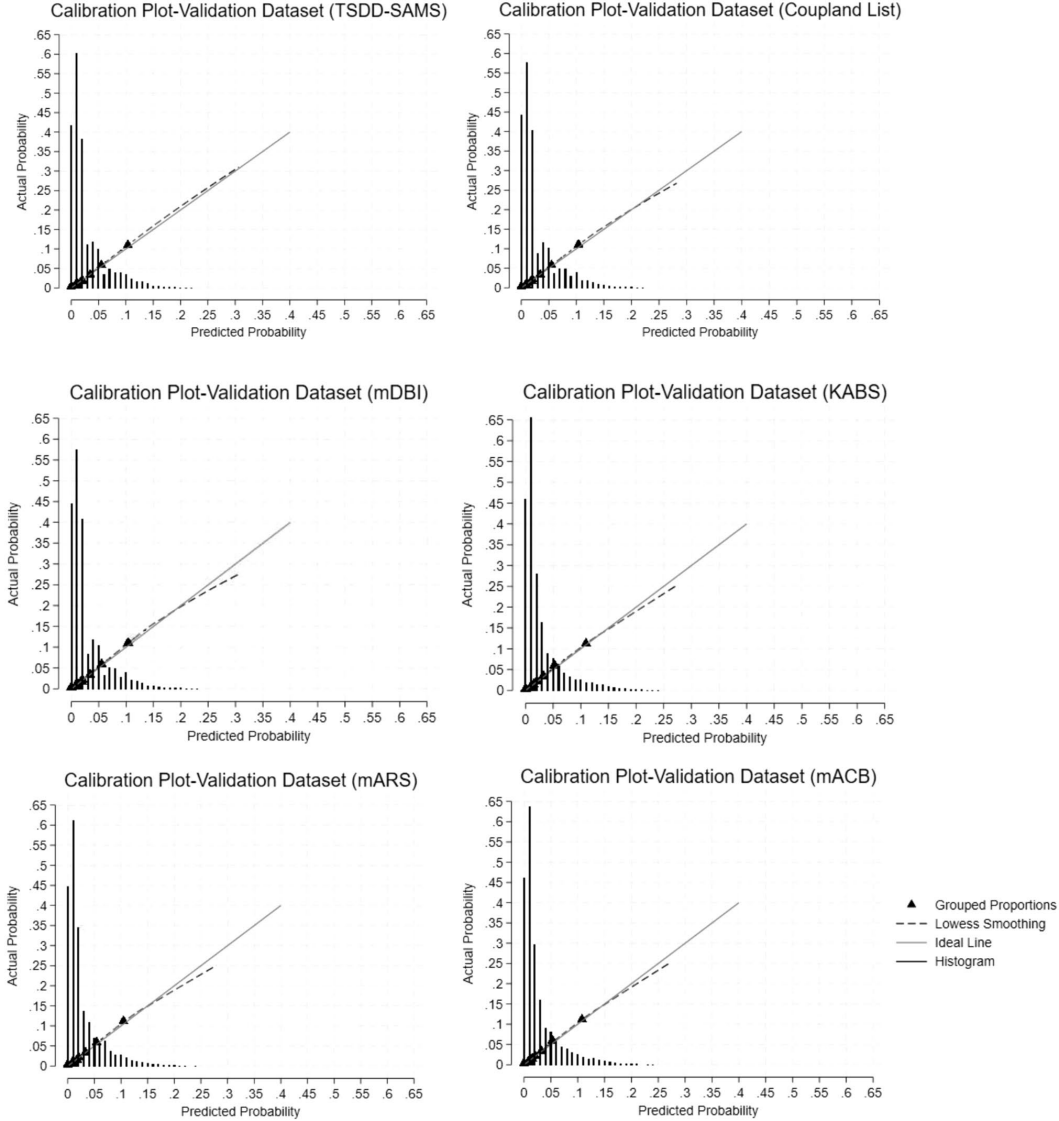
Calibration plots for cognitive impairment hospitalisations/ED visits related to anticholinergic adverse outcomes in validation dataset across scales. Calibration plots showing predicted versus observed probabilities for cognitive impairment outcome across scales in the validation cohorts. The diagonal line represents perfect calibration; the smoothed line (lowess) shows model performance, with histograms indicating the distribution of predicted probabilities

Figure 2 shows the calibration plots for the cognitive impairment models across scales in the temporal validation cohort. Predicted and observed risks were closely aligned over most of the probability range, with slight overestimation at higher predicted probabilities. The vertical histograms indicate that most predicted probabilities were concentrated below 0.2, consistent with the low absolute event risk in the study population.

### Absolute predicted risk across anticholinergic burden deciles

Table 4 presents predicted probabilities for cognitive impairment across scale-specific anticholinergic burden deciles. Predicted risk increased across higher exposure deciles, though magnitude varied by scale. In the highest decile, predicted risk ranged from 5.27% for TSDD-SAMS to 7.58% for KABS. These findings show higher anticholinergic burden was associated with higher predicted risk, while highlighting variation in risk gradients across scales.

**Table 4:** Predicted probabilities across increasing anticholinergic burden levels for cognitive impairment-related hospitalisation/ED visits by scale

Group decile	TSDD-SAMS		Coupland List		mDBI	
	Score range	Predicted risk % (95% CI)	Score range	Predicted risk % (95% CI)	Score range	Predicted risk % (95% CI)
0	0	2.42 (2.41–2.43)	0	2.36 (2.35–2.37)	0	2.37 (2.36–2.38)
1	0.5–8	4.61 (4.36–4.87)	0.5–8	5.41 (5.15–5.66)	0.00–0.03	3.59 (3.31–3.87)
2	8.33–24.67	2.45 (2.40–2.50)	8.33–48.33	2.60 (2.55–2.65)	0.03–0.04	2.48 (2.43–2.54)
3	25–48.33	3.48 (3.32–3.65)	50–81	3.49 (3.38–3.60)	0.04–0.12	3.04 (2.95–3.14)
4	50–111.33	3.58 (3.49–3.66)	83.33–148	4.50 (4.37–4.64)	0.13–0.28	3.57 (3.46–3.67)
5	112–248.33	4.26 (4.12–4.39)	150–299.17	4.37 (4.24–4.50)	0.28–0.87	4.39 (4.28–4.51)
6	250–399.33	4.22 (4.09–4.34)	300–418.67	4.09 (3.98–4.20)	0.87–2.03	4.07 (3.95–4.19)
7	400–693.33	4.44 (4.31–4.57)	420–716.67	4.93 (4.78–5.07)	2.03 – 3.42	4.42 (4.28–4.55)
8	700–1047	4.26 (4.13–4.39)	720–1233.33	4.73 (4.59–4.86)	3.43–4.97	4.34 (4.22–4.46)
9	1050 – 18,000	5.27 (5.11–5.44)	1235–27,360	5.48 (5.33–5.64)	4.97–7.45	4.52 (4.39–4.65)
10					7.45–111.03	6.05 (5.89–6.22)

Group decile	KABS		mARS		mACB	
	Score range	Predicted risk % (95% CI)	Score range	Predicted risk % (95% CI)	Score range	Predicted Risk % (95% CI)
0	0	1.31 (1.30–1.31)	0	1.96 (1.95–1.97)	0	1.22 (1.21–1.22)
1	0.00–0.03	2.35 (2.30–2.39)	0.00–0.03	5.93 (5.73–6.14)	0.00–0.06	1.85 (1.82–1.88)
2	0.03–0.08	1.96 (1.93–1.99)	0.03–0.05	3.05 (2.99–3.12)	0.06–0.15	2.12 (2.09–2.15)
3	0.08–0.21	2.85 (2.81–2.90)	0.06–0.09	2.91 (2.85–2.97)	0.15–0.27	2.42 (2.38–2.45)
4	0.21–0.31	2.91 (2.87–2.96)	0.09–0.15	4.36 (4.27–4.45)	0.27–0.41	2.99 (2.94–3.02)
5	0.31–0.53	3.87 (3.81–3.92)	0.15–0.27	4.69 (4.59–4.79)	0.41–0.60	3.47 (3.42–3.51)
6	0.53–0.82	4.56 (4.49–4.62)	0.27–0.41	5.36 (5.25–5.48)	0.60–0.82	3.24 (3.20–3.29)
7	0.82–1.10	4.04 (3.98–4.10)	0.41–0.59	5.01 (4.92–5.11)	0.82–1.11	3.98 (3.93–4.03)
8	1.10 – 1.66	5.52 (5.45–5.60)	0.59–0.99	5.63 (5.51–5.74)	1.11–1.67	4.91 (4.85–4.97)
9	1.66 – 2.64	5.59 (5.51–5.67)	0.99–1.51	5.22 (5.12–5.33)	1.67–2.71	5.56 (5.49–5.63)
10	2.64–34.18	7.58 (7.49–7.67)	1.51–1134.25	6.13 (6.00–6.26)	2.71–36.32	7.17 (7.09–7.26)

Score ranges were based on scale-specific decile cut-points among participants with positive exposure; score = 0 was retained as a separate no-exposure category. For some scales, adjacent cut-points coincided, resulting in fewer than ten positive-exposure groups

## Discussion

This study showed that predictive performance varied across anticholinergic burden scales, with KABS generally showing slightly higher and more consistent performance than the other scales. Cognitive impairment showed the highest discrimination across scales and was taken forward for internal and temporal validation. Predicted risks increased across higher anticholinergic burden deciles.

Few previous studies have reported both internal and external validation of prediction models using anticholinergic burden scales. One assessed the Clinician-Rated Anticholinergic Scale (CrAS) for delirium-related hospitalisations in older inpatients without cross-scale comparisons [44], while another compared multiple scales for predicting falls in a primary care population using self-reported data prone to recall bias [23]. This study adds a population-based comparison of six validated scales across several hospitalisation and ED outcomes using a consistent modelling framework, and temporal validation in a later cohort. The focus was comparative evaluation of scale performance rather than development of a clinical prediction model for routine clinical use.

KABS showed the highest c-statistics across outcomes, although absolute differences from other scales were small. Cognitive impairment showed the highest discrimination, with temporal validation c-statistics of 0.795–0.806 across scales, indicating acceptable to excellent performance [45], while other outcomes showed lower but acceptable performance. These discrimination levels are notable because hospitalisation and ED outcomes in older adults are multifactorial and not solely explained by anticholinergic burden. Previous studies reported c-statistics around 0.7 for falls [23] and 0.5–0.7 for peripheral and central adverse effects [19, 22, 46]. The models performed at least as well as, and sometimes better than, comparable tools. This may reflect including scales that performed well in earlier comparative work [11], although differences in populations, outcomes, and modelling approaches across studies warrant consideration.

Performance differences may reflect variations in scale development, scoring algorithms, and medication coverage. KABS, mACB, and mARS include potency and dose in burden calculations, while TSDD-SAMS, mDBI, and Coupland List consider doses without potency. Including potency may improve sensitivity to clinical exposures. All scales (except Coupland List) were developed through expert consensus and have more validation than approaches based on pharmacological activity. KABS combines published scales, uses a two-round Delphi process to resolve discrepancies, and adds locally available medications not previously rated. KABS and mARS have been validated for peripheral and central adverse outcomes, contributing to their stronger performance. KABS uses WHO-defined daily doses to standardise calculations. The higher prevalence by mACB (51.9–53.6%), KABS (44.1–45.5%), mARS (22.5–23.2%) compared to other scales suggests broader medication inclusion may add predictive information beyond demographic and clinical covariates.

Internal bootstrap validation quantified and adjusted potential optimism, providing more reliable estimates than apparent performance alone [16, 42]. Temporal validation in a 2017–18 cohort tested reproducibility between periods, providing better assessment of generalisability than random split-sample approaches [15]. Internal and temporal validation showed little evidence of performance deterioration across scales. KABS showed the highest discrimination, though differences between scales remained modest, and calibration measures were similar, with slopes close to 1.0 and calibration-in-the-large close to zero. One study [23] reported a lower post-validation calibration slope of 0.716 for falls, possibly reflecting its narrower, higher-risk primary care cohort (≥ 3 chronic conditions) and reliance on self-reported medication data. The broader, whole-population administrative dataset used here may have contributed to the more stable calibration observed.

While calibration metrics showed good agreement, calibration plots revealed minor deviations at higher predicted probabilities, with curves below the ideal line, suggesting modest overestimation for highest-risk individuals. This pattern is unsurprising, as few participants had predicted probabilities above 20%, where estimates were less stable. Most predicted probabilities were concentrated at lower values (2–15%), where calibration remained strong. This tendency towards overprediction at the upper end indicates caution when interpreting predicted probabilities in high-risk patients, as this may slightly overstate individual risk at the highest probabilities. Predicted probabilities showed a gradient across anticholinergic burden deciles, though the magnitude varied between scales. For cognitive impairment, predicted risk in the highest decile ranged from 5.27% for TSDD-SAMS to 7.58% for KABS. These findings indicate that higher anticholinergic burden was linked to progressively higher predicted risk. Although risks remained modest, cognitive impairment is clinically important in older adults, and these estimates may support risk stratification and medication review prioritization [47, 48].

### Strengths and limitations

A key strength was using a large, representative cohort of older adults to compare scales across important outcomes. Temporal validation assessed stability across periods and translating scores into absolute risks provided interpretable links between modelling and clinical application. The study extends prior work by evaluating predictive performance rather than associations alone.

Limitations include reliance on PBS dispensing data, which does not capture over-the-counter medications (for example cold, flu and allergy medications) or confirm adherence, potentially affecting comparative performance across scales based on how each scale includes medications obtained over the counter. Outcome definitions using hospital and ED diagnosis codes served as proxies for anticholinergic adverse effects, but these codes do not confirm causality and may include unrelated events, reducing specificity [49]. Hospital and ED records capture only events severe enough to require acute care, so milder events managed in community/primary care may not be represented, reducing sensitivity. The composite primary outcome combined clinically heterogeneous events, which may have diluted associations despite its usefulness as a summary measure of overall acute anticholinergic-related presentations. Additionally, temporal validation was done for cognitive impairment models only, so performance stability over time was not assessed for other outcomes. Although KABS was developed in similar administrative claims, differences in prescription patterns between Korea and Australia could influence performance. These factors should be considered when interpreting transferability beyond the present setting.

### Implications for practice and research

These findings suggest the KABS may be useful for integration into prescribing support tools and medication review protocols for older adults. Given the small differences between scales, these findings should not be the sole basis for preferring one scale in practice, and any medication review should still take into account individual patient factors such as frailty, comorbidity, renal function, and polypharmacy [50], which may modify susceptibility to adverse effects. Future research should validate model performance in different settings and longer time periods, examine whether combining burden scales with dynamic clinical factors improves prediction, and assess whether integrating such tools into routine care affects prescribing behaviour or patient outcomes.

## Conclusion

This study compared six anticholinergic burden scales, evaluated their predictive performance across hospitalisation and ED outcomes. KABS demonstrated the most consistent performance, particularly for cognitive impairment, although differences between scales were modest. Presenting absolute predicted probabilities may support medication review by identifying individuals at relatively higher risk of anticholinergic-related adverse outcomes.

## Supplementary Information

Below is the link to the electronic supplementary material.

Supplementary file 1 (DOCX 948 KB)

## Data Availability

The data that support the findings of this study are available from the relevant data custodians of the study data sets. Restrictions by the data custodians mean that the data are not publicly available or able to be provided by the authors. Researchers wishing to access the data sets used in this study should refer to the Western Australian Data Linkage Unit and the Australian Institute of Health and Welfare.
