# Supplementary material for: Comparative predictive performance of anticholinergic burden scales for anticholinergic-related hospitalisation and emergency department visits in older adults: a population-based study of model development and temporal validation
Source: Int J Clin Pharm. 2026 Apr 17;48(4):1646–59. doi: 10.1007/s11096-026-02145-9 (PMC13369043; doi:10.1007/s11096-026-02145-9)
Supplement: Supplementary file 1 — (DOCX 948 KB) [file 11096_2026_2145_MOESM1_ESM.docx]

**Title: Comparative Predictive Performance of Anticholinergic Burden Scales for Anticholinergic-Related Hospitalisation and Emergency Department Visits in Older Adults: A Population-Based Study of Model Development and Temporal Validation**

**International Journal of Clinical Pharmacy**

Valentina M Srikartika^1, 2^, David Youens^1^, Rachael Moorin^1,3^, Ninh Ha^1^

^1^ Health Economics and Data Analytics, School of Population Health, Curtin University, Perth, Western Australia 6102, Australia

^2^ Pharmacy Program Study, Faculty of Mathematics and Natural Science, Lambung Mangkurat University, Banjarbaru, South Kalimantan 70714, Indonesia

^3^ School of Population and Global Health, The University of Western Australia, Crawley, Western Australia, 6001, Australia

**Corresponding author:**E-mail address: valentina.srikartika@curtin.edu.au (Valentina M Srikartika)

**Suppl 1. Flow diagram of annual cohort construction and panel couplet design for exposure and outcome ascertainment**


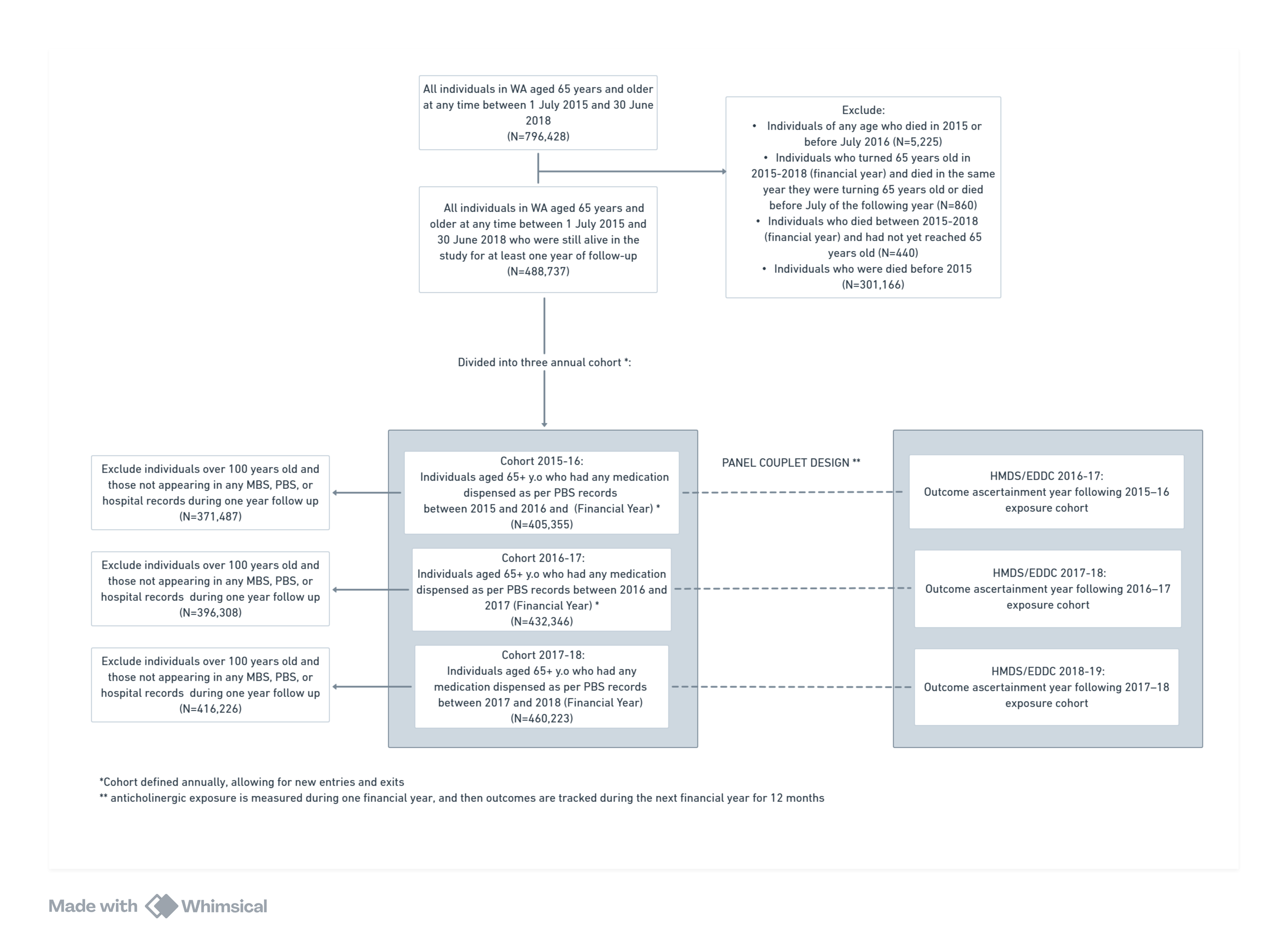


**Suppl 2.** **Description of Scoring Process for Calculating Cumulative Anticholinergic Burden by Various Scales**

1. The Total Standardised Daily Doses-Summated Anticholinergic Medications Scale (TSDD-SAMS)

Introduced by Gray et al. in 2015 [1], the TSDD measure utilises the Summated Anticholinergic Medications Scale (SAMS) medication list. This list comprises medications known for their strong anticholinergic activity, accompanied by each medication's recommended minimum effective daily dose. To compute this measure, specific medication use variables are essential: the medication name, strength, route of administration, date dispensed, and the quantity dispensed for each anticholinergic medication. The steps to determine the cumulative anticholinergic exposure as the total standardized daily dose are as follows:

1. Determine the total anticholinergic medication dose for each dispensed prescription by multiplying the tablet's strength by the number of tablets dispensed.
2. Compute the Standardised Daily Dose (SDD) for each dispensed prescription: This involves dividing the estimated total anticholinergic medication dose (from step a) by the recommended minimum effective daily dose for older adults, as outlined in the SAMS medication list.
3. Calculate the total standardized daily dose (TSDD) for each participant by summing the SDD (from step b) for all dispensed anticholinergic prescriptions throughout the study or exposure period.
4. Categorization of the Total Score for Each Participant: Scores were categorized as follows: no use, 1–90, 91–365, 366–1095, and > 1095 (i.e., >3 years). The categorization cutoff points were determined based on clinical interpretability and exposure distribution observed within the study sample.
5. The Modified Drug Burden Index

Introduced by Bryne et al. in 2018 [2], the modified DBI used the medication list that was formulated using previously published studies, drug monographs, and expert opinions. A list of anticholinergic medications, accompanied by their respective minimum effective daily doses, was curated. The cumulative anticholinergic exposure is determined using the equation:

$$DBI=\sum\frac{D}{\delta+D}$$

In this equation, D represents the daily dose consumed by the patient, while δ denotes the minimum effective daily dose for a specific anticholinergic medication, as identified in the study's anticholinergic medication list.

To compute the estimated daily dose of anticholinergic medication, the strength of the medication was multiplied by the total quantity dispensed during the study or the exposure period. This total dose was then standardized into a daily dose by dividing it by the duration of the study or exposure.

1. The Modified Anticholinergic Cognitive Burden

Introduced by Ah Young et al. in 2019 [3], the modified ACB quantifies the cumulative exposure to anticholinergic medications. This measure employs an updated list of recognised medications with anticholinergic potency as per the ACB scale [4]. Notably, it also includes anticholinergic medications available in the study's country but not previously listed in the ACB scale.

For each dispensed anticholinergic medication, the prescribed dose was adjusted in line with the Defined Daily Dose (DDD) as stipulated by the WHO. To determine the average daily anticholinergic score over the three-month study duration for each patient, the following equation was employed:

$$Average daily ACB Score=\frac{\sum_{i=1}^{n} \frac{Total prescribed dose of Ai during period x ACB score of Ai}{WHO-DDD of Ai}}{study or exposure duration}$$

Where A represents the i^th^ anticholinergic agent prescribed to a patient, with i ranging from 1 to n; ACB stands for the Anticholinergic Cognitive Burden potency score; WHO−DDD denotes the defined daily doses for a specific anticholinergic medication as established by the WHO

1. The Modified Anticholinergic Risk Scale

Developed in 2019 by Hwang et al. [5], this anticholinergic burden measure utilizes the list of anticholinergic medications from the ARS scale [6]. A notable modification in this measure is the inclusion of anticholinergic medications that were available in the study's country but not listed in the original ARS scale.

The cumulative anticholinergic burden for a patient, as defined by this measure, is computed as the sum of the total burden from all prescribed anticholinergic medications. This is represented by the following equation:

$$Average daily ARS Score=\frac{\sum_{i=1}^{n} \frac{Total prescribed dose of Ai during period x ARS score of Ai}{WHO-DDD of Ai}}{study or exposure duration}$$

Where A represents the i th anticholinergic medication prescribed to a patient, with i ranging from 1 to n; ARS stands for the potency score as defined by the Anticholinergic Risk Scale; WHO−DDD denotes the defined daily dose as established by the WHO. Study or exposure duration was employed to standardize the daily exposure in the measure.

In terms of categorization, an average daily ARS score of ≥ 2 was considered indicative of a high anticholinergic burden. However, it is worth noting that there is no clinical consensus on the optimal cutoff for this measure.

1. The Anticholinergic Score by Coupland

The Coupland list, introduced in 2019 by Coupland et al [7], is a modified approach to calculating the cumulative exposure of anticholinergic medications. It builds upon the methodology developed by Gray [1], known as the TSDD measure.

The list of anticholinergic medications used in the TSDD was modified. This involved integrating anticholinergic medication lists from the updated Beers Criteria, ACB scale, and Duran’s list. Additionally, medications known for their significant anticholinergic properties, as cited in the British National Formulary (BNF) Drug Reference Handbook, were included. The methodology for determining the cumulative exposure mirrors that of the TSDD measurement. The total dose of each prescription was calculated by multiplying the number of tablets prescribed by the dose per tablet. This is also applicable to other forms such as solutions, inhalers, injections, or patches. Subsequently, these values were divided by the minimum effective daily dose values recommended for older adults, yielding a number of standardized daily doses for each prescription.

A distinguishing feature of the Coupland list, aside from the expanded list of recognized anticholinergic medications, is the source of recommendations for the minimum effective daily dose for older adults. This study relies on recommendations from both Lexicomp and BNF.

1. Korean Anticholinergic Burden Scale (KABS).

Introduced by Hwang et al. in 2021 [8], the KABS was developed to provide a country-specific tool for quantifying cumulative anticholinergic exposure, incorporating medicines commonly used in Korea that were not captured on earlier international scales. The medication list was compiled through a review of multiple existing anticholinergic rating systems, including the Anticholinergic Cognitive Burden (ACB) scale, Anticholinergic Risk Scale (ARS), Anticholinergic Drug Scale (ADS), Anticholinergic Activity Scale (AAS), Beers Criteria, and the “strong anticholinergics” list by Gray et al., with ratings harmonised to a 0–3 scoring system. Newly identified medicines with anticholinergic properties available in Korea were added based on an expert consensus.

To calculate the KABS for each individual:

1. Identify all dispensed anticholinergic medicines during the study or exposure period using the KABS medication list.
2. Assign an anticholinergic potency score (0 = no effect, 1 = mild, 2 = moderate, and 3 = strong) for each medicine based on the KABS list.
3. The daily anticholinergic burden for each medicine was calculated by multiplying the potency score by the proportion of the Defined Daily Dose (DDD, WHO ATC index) dispensed.
   - - For each dispensing, the total dose = (strength per unit × quantity dispensed).
     - Standardised daily dose = (total dose ÷ WHO-DDD).
4. Sum across all medicines for each day to obtain the total daily KABS score.
5. Average daily KABS score over the exposure period ( 12 months) = (Σ daily scores) ÷ (number of days in the period).

**Suppl 3. Full code lists and outcome classification rules**

| **Description** | **Diagnosis Code (ICD 10)** |
| --- | --- |
| Fall | W00-W19 |
| Fracture | S02,S12,S22,S32,S42,S52,S62,S72,S82,S92,T02,T08,T10,T12, T14.2 |
| Dizziness | R42 |
| Delirium | F05 |
| Constipation | K59.0 |
| Urinary Retention | R33, R39.1 |
| Disorientation, unspecified | R41.0 |
| Restless & Agitation | R45.1 |
| Mild cognitive disorder | F06.7 |

**Re-categorisation of the outcome**

| **Diagnosis** | **Study Outcomes** | **Composite Endpoint (primary outcome)** |
| --- | --- | --- |
| Fall | Fall, fractures, & dizziness | Any hospitalisation/ ED visit related to anticholinergic adverse effects |
| Fracture |  |  |
| Dizziness |  |  |
| Delirium | Cognitive impairment |  |
| Disorientation, unspecified |  |  |
| Restless & Agitation |  |  |
| Mild cognitive disorder |  |  |
| Constipation | Constipation & urinary retention |  |
| Urinary Retention |  |  |

**Suppl 4. Final Model Specifications (stata syntax)**

*** Any anticholinergic-related hospitalisation/ED visit**

*logit any_ae i.age_cat##i.sex i.age_cat##i.maccs ///*

*i.maccs##i.sex i.aria i.seifa*

*** Falls, fractures, or dizziness**

*logit ae_ffd i.age_cat##i.sex i.age_cat##i.maccs///*

*i.maccs ##i.sex i.aria i.seifa*

*** Cognitive impairment**

*logit ae_ci i.age_cat##i.sex i.age_cat##i.maccs///*

*i.maccs ##i.sex i.aria i.seifa*

*** Constipation or urinary retention**

*logit ae_cur i.age_cat##i.sex i.age_cat##i.maccs///*

*i.maccs ##i.sex i.aria i.seifa*

**Notes:**

**any_ae: any_adverse_effects / ae_ffd : fall_fractures_dizziness_event / ae_ci : cognitive_impairment_event / ae_cur: constipation_urinary retention_event:** Binary outcome variables indicating whether the individual experienced the respective anticholinergic-related hospitalisation/ED visit during the study period.

**age_cat:** Categorical age variable

**sex:** Binary variable for biological sex

**maccs:** Multipurpose Australian Comorbidity Scoring System

**aria:** Accessibility/Remoteness Index of Australia

**seifa:** Socio-Economic Index for Areas Index of Relative Socio-economic Disadvantage

**Suppl 5. Baseline characteristics of the development and validation cohorts for the other outcome**

| **Fall, Fractures, and Dizziness** | | | | | | | | | | | | | | | | |
| --- | --- | --- | --- | --- | --- | --- | --- | --- | --- | --- | --- | --- | --- | --- | --- | --- |
| **Variables** | **Categories/ Unit** | **Development set 2015-16 (n= 323,682)** | | | | | **Development set 2016-17 (n= 334,304)** | | | | | **Validation set (n=330,684)** | | | | |
|  |  | **No Hosp/ED (n,%)** | | **Yes Hosp/ED (n,%)** | | **p value** | **No Hosp/ED (n,%)** | | **Yes Hosp/ED (n,%)** | | **p value** | **No Hosp/ED (n,%)** | | **Yes Hosp/ED (n,%)** | | **p value** |
| Sex | Female | 160,076 | 93.5 | 11,057 | 6.5 | <0.001 | 164,872 | 93.4 | 11,582 | 6.6 | <0.001 | 163,613 | 93.2 | 11,928 | 6.8 | <0.001 |
|  | Male | 145,493 | 95.4 | 7,056 | 4.6 |  | 150,318 | 95.2 | 7,532 | 4.8 |  | 146,864 | 94.7 | 8,279 | 5.3 |  |
|  |  |  |  |  |  |  |  |  |  |  |  |  |  |  |  |  |
| Age | 65-74 | 184,228 | 97.1 | 5,571 | 2.9 | <0.001 | 190,336 | 97.0 | 5,839 | 3.0 | <0.001 | 184,182 | 96.8 | 6,171 | 3.2 | <0.001 |
|  | 75-84 | 90,237 | 93.0 | 6,829 | 7.0 |  | 93,195 | 92.8 | 7,205 | 7.2 |  | 94,086 | 92.5 | 7,651 | 7.5 |  |
|  | 85-94 | 29,301 | 84.9 | 5,233 | 15.2 |  | 29,758 | 84.4 | 5,496 | 15.6 |  | 30,169 | 83.9 | 5,803 | 16.1 |  |
|  | 95+ | 1,803 | 79.0 | 480 | 21.0 |  | 1,901 | 76.8 | 574 | 23.2 |  | 2,040 | 77.8 | 582 | 22.2 |  |
|  |  |  |  |  |  |  |  |  |  |  |  |  |  |  |  |  |
| SEIFA | Least disadvantage | 88,251 | 94.3 | 5,364 | 5.7 | <0.001 | 91,330 | 94.2 | 5,604 | 5.8 | <0.001 | 89,965 | 94.0 | 5,771 | 6.0 | <0.001 |
|  | Less disadvantage | 47,072 | 94.1 | 2,977 | 6.0 |  | 48,351 | 93.7 | 3,250 | 6.3 |  | 47,788 | 93.3 | 3,413 | 6.7 |  |
|  | Moderate disadvantage | 54,911 | 94.5 | 3,198 | 5.5 |  | 56,750 | 94.4 | 3,365 | 5.6 |  | 56,032 | 93.9 | 3,626 | 6.1 |  |
|  | High disadvantage | 81,738 | 94.9 | 4,414 | 5.1 |  | 84,265 | 94.8 | 4,669 | 5.3 |  | 83,063 | 94.4 | 4,975 | 5.7 |  |
|  | Highest disadvantage | 33,351 | 93.9 | 2,152 | 6.1 |  | 34,218 | 93.9 | 2,223 | 6.1 |  | 33,366 | 93.3 | 2,415 | 6.8 |  |
|  |  |  |  |  |  |  |  |  |  |  |  |  |  |  |  |  |
| ARIA | Major cities | 225,722 | 93.9 | 14,566 | 6.1 | <0.001 | 232,331 | 93.8 | 15,258 | 6.2 | <0.001 | 229,204 | 93.5 | 15,981 | 6.5 | <0.001 |
|  | Inner regional | 43,187 | 95.9 | 1,858 | 4.1 |  | 44,841 | 95.7 | 2,039 | 4.4 |  | 44,405 | 95.3 | 2,177 | 4.7 |  |
|  | Outer regional | 26,871 | 95.5 | 1,267 | 4.5 |  | 27,831 | 95.5 | 1,308 | 4.5 |  | 27,251 | 94.8 | 1,500 | 5.2 |  |
|  | Remote | 5,651 | 95.8 | 245 | 4.2 |  | 5,816 | 95.1 | 298 | 4.9 |  | 5,714 | 94.8 | 313 | 5.2 |  |
|  | Very Remote | 4,128 | 95.9 | 176 | 4.1 |  | 4,360 | 95.4 | 211 | 4.6 |  | 3,896 | 94.3 | 234 | 5.7 |  |
|  |  |  |  |  |  |  |  |  |  |  |  |  |  |  |  |  |
| MACCS | 0 | 71,617 | 96.9 | 2,269 | 3.1 | <0.001 | 72,354 | 97.1 | 2,167 | 2.9 | <0.001 | 65,822 | 96.8 | 2,149 | 3.2 | <0.001 |
|  | 1-4 | 141,569 | 95.9 | 6,031 | 4.1 |  | 145,274 | 95.9 | 6,252 | 4.1 |  | 142,273 | 95.6 | 6,495 | 4.4 |  |
|  | 5-9 | 77,504 | 91.8 | 6,888 | 8.2 |  | 81,456 | 91.7 | 7,384 | 8.3 |  | 84,876 | 91.7 | 7,735 | 8.4 |  |
|  | 10+ | 14,879 | 83.6 | 2,925 | 16.4 |  | 16,106 | 83.0 | 3,311 | 17.1 |  | 17,506 | 82.1 | 3,828 | 17.9 |  |
|  |  |  |  |  |  |  |  |  |  |  |  |  |  |  |  |  |
|  |  |  |  |  |  |  |  |  |  |  |  |  |  |  |  |  |
| **Cognitive Impairment** | | | | | | | | | | | | | | | | |
| **Variables** | **Categories/ Unit** | **Development set 2015-16 (n= 323,682)** | | | | | **Development set 2016-17 (n= 334,304)** | | | | | **Validation set (n=330,684)** | | | | |
|  |  | **No Hosp/ED (n,%)** | | **Yes Hosp/ED (n,%)** | | **p value** | **No Hosp/ED (n,%)** | | **Yes Hosp/ED (n,%)** | | **p value** | **No Hosp/ED (n,%)** | | **Yes Hosp/ED (n,%)** | | **p value** |
| Sex | Female | 167,101 | 97.6 | 4,032 | 2.4 | <0.001 | 172,331 | 97.7 | 4,123 | 2.3 | <0.001 | 171,091 | 97.5 | 4,450 | 2.5 | 0.015 |
|  | Male | 148,465 | 97.3 | 4,084 | 2.7 |  | 153,738 | 97.4 | 4,112 | 2.6 |  | 150,675 | 97.1 | 4,468 | 2.9 |  |
|  |  |  |  |  |  |  |  |  |  |  |  |  |  |  |  |  |
| Age | 65-74 | 187,931 | 99.0 | 1,868 | 1.0 | <0.001 | 194,414 | 99.1 | 1,761 | 0.9 | <0.001 | 188,465 | 99.0 | 1,888 | 1.0 | <0.001 |
|  | 75-84 | 93,905 | 96.7 | 3,161 | 3.3 |  | 97,085 | 96.7 | 3,315 | 3.3 |  | 98,187 | 96.5 | 3,550 | 3.5 |  |
|  | 85-94 | 31,706 | 91.8 | 2,828 | 8.2 |  | 32,391 | 91.9 | 2,863 | 8.1 |  | 32,791 | 91.2 | 3,181 | 8.8 |  |
|  | 95+ | 2,024 | 88.7 | 259 | 11.3 |  | 2,179 | 88.0 | 296 | 12.0 |  | 2,323 | 88.6 | 299 | 11.4 |  |
|  |  |  |  |  |  |  |  |  |  |  |  |  |  |  |  |  |
| SEIFA | Least disadvantage | 91,334 | 97.6 | 2,281 | 2.4 | <0.001 | 94,588 | 97.6 | 2,346 | 2.4 | <0.001 | 93,277 | 97.4 | 2,459 | 2.6 | <0.001 |
|  | Less disadvantage | 48,609 | 97.1 | 1,440 | 2.9 |  | 50,159 | 97.2 | 1,442 | 2.8 |  | 49,663 | 97.0 | 1,538 | 3.0 |  |
|  | Moderate disadvantage | 56,703 | 97.6 | 1,406 | 2.4 |  | 58,644 | 97.6 | 1,471 | 2.5 |  | 58,026 | 97.3 | 1,632 | 2.7 |  |
|  | High disadvantage | 84,066 | 97.6 | 2,086 | 2.4 |  | 86,857 | 97.7 | 2,077 | 2.3 |  | 85,742 | 97.4 | 2,296 | 2.6 |  |
|  | Highest disadvantage | 34,605 | 97.5 | 898 | 2.5 |  | 35,543 | 97.5 | 898 | 2.5 |  | 34,793 | 97.2 | 988 | 2.8 |  |
|  |  |  |  |  |  |  |  |  |  |  |  |  |  |  |  |  |
| ARIA | Major cities | 233,800 | 97.3 | 6,488 | 2.7 | <0.001 | 241,095 | 97.4 | 6,494 | 2.6 | <0.001 | 238,129 | 97.1 | 7,056 | 2.9 | <0.001 |
|  | Inner regional | 44,183 | 98.1 | 862 | 1.9 |  | 45,907 | 97.9 | 973 | 2.1 |  | 45,542 | 97.8 | 1,040 | 2.2 |  |
|  | Outer regional | 27,569 | 98.0 | 569 | 2.0 |  | 28,564 | 98.0 | 575 | 2.0 |  | 28,158 | 97.9 | 593 | 2.1 |  |
|  | Remote | 5,791 | 98.2 | 105 | 1.8 |  | 6,011 | 98.3 | 103 | 1.7 |  | 5,907 | 98.0 | 120 | 2.0 |  |
|  | Very Remote | 4,212 | 97.9 | 92 | 2.1 |  | 4,481 | 98.0 | 90 | 2.0 |  | 4,021 | 97.4 | 109 | 2.6 |  |
|  |  |  |  |  |  |  |  |  |  |  |  |  |  |  |  |  |
| MACCS | 0 | 72,999 | 98.8 | 887 | 1.2 | <0.001 | 73,743 | 99.0 | 778 | 1.0 | <0.001 | 67,180 | 98.8 | 791 | 1.2 | <0.001 |
|  | 1-4 | 145,413 | 98.5 | 2,187 | 1.5 |  | 149,333 | 98.6 | 2,193 | 1.5 |  | 146,470 | 98.5 | 2,298 | 1.5 |  |
|  | 5-9 | 81,143 | 96.2 | 3,249 | 3.9 |  | 85,567 | 96.3 | 3,273 | 3.7 |  | 89,218 | 96.3 | 3,393 | 3.7 |  |
|  | 10+ | 16,011 | 89.9 | 1,793 | 10.1 |  | 17,426 | 89.8 | 1,991 | 10.3 |  | 18,898 | 88.6 | 2,436 | 11.4 |  |
|  |  |  |  |  |  |  |  |  |  |  |  |  |  |  |  |  |
|  |  |  |  |  |  |  |  |  |  |  |  |  |  |  |  |  |
| **Constipation and Urinary Retention** | | | | | | | | | | | | | | | | |
| **Variables** | **Categories/**  **Unit** | **Development set 2015-16 (n= 323,682)** | | | | | **Development set 2016-17 (n= 334,304)** | | | | | **Validation set (n=330,684)** | | | | |
|  |  | **No Hosp/ED (n,%)** | | **Yes Hosp/ED (n,%)** | | **p value** | **No Hosp/ED (n,%)** | | **Yes Hosp/ED (n,%)** | | **p value** | **No Hosp/ED (n,%)** | | **Yes Hosp/ED (n,%)** | | **p value** |
| Sex | Female | 163,845 | 95.7 | 7,288 | 4.3 | <0.001 | 168,639 | 95.6 | 7,815 | 4.4 | <0.001 | 167,722 | 95.6 | 7,819 | 4.5 | 0.015 |
|  | Male | 144,672 | 94.8 | 7,877 | 5.2 |  | 149,530 | 94.7 | 8,320 | 5.3 |  | 146,674 | 94.5 | 8,469 | 5.5 |  |
|  |  |  |  |  |  |  |  |  |  |  |  |  |  |  |  |  |
| Age | 65-74 | 184,735 | 97.3 | 5,064 | 2.7 | <0.001 | 190,798 | 97.3 | 5,377 | 2.7 | <0.001 | 184,857 | 97.1 | 5,496 | 2.9 | <0.001 |
|  | 75-84 | 91,190 | 94.0 | 5,876 | 6.1 |  | 94,114 | 93.7 | 6,286 | 6.3 |  | 95,409 | 93.8 | 6,328 | 6.2 |  |
|  | 85-94 | 30,597 | 88.6 | 3,937 | 11.4 |  | 31,126 | 88.3 | 4,128 | 11.7 |  | 31,847 | 88.5 | 4,125 | 11.5 |  |
|  | 95+ | 1,995 | 87.4 | 288 | 12.6 |  | 2,131 | 86.1 | 344 | 13.9 |  | 2,283 | 87.1 | 339 | 12.9 |  |
|  |  |  |  |  |  |  |  |  |  |  |  |  |  |  |  |  |
| SEIFA | Least disadvantage | 89,301 | 95.4 | 4,314 | 4.6 | 0.089 | 92,381 | 95.3 | 4,553 | 4.7 | <0.001 | 91,308 | 95.4 | 4,428 | 4.6 | <0.001 |
|  | Less disadvantage | 47,618 | 95.1 | 2,431 | 4.9 |  | 48,865 | 94.7 | 2,736 | 5.3 |  | 48,499 | 94.7 | 2,702 | 5.3 |  |
|  | Moderate disadvantage | 55,355 | 95.3 | 2,754 | 4.7 |  | 57,290 | 95.3 | 2,825 | 4.7 |  | 56,787 | 95.2 | 2,871 | 4.8 |  |
|  | High disadvantage | 82,194 | 95.4 | 3,958 | 4.6 |  | 84,740 | 95.3 | 4,194 | 4.7 |  | 83,620 | 95.0 | 4,418 | 5.0 |  |
|  | Highest disadvantage | 33,802 | 95.2 | 1,701 | 4.8 |  | 34,617 | 95.0 | 1,824 | 5.0 |  | 33,925 | 94.8 | 1,856 | 5.2 |  |
|  |  |  |  |  |  |  |  |  |  |  |  |  |  |  |  |  |
| ARIA | Major cities | 228,482 | 95.1 | 11,806 | 4.9 | <0.001 | 235,059 | 94.9 | 12,530 | 5.1 | <0.001 | 232,645 | 94.9 | 12,540 | 5.1 | <0.001 |
|  | Inner regional | 43,328 | 96.2 | 1,717 | 3.8 |  | 44,981 | 96.0 | 1,899 | 4.1 |  | 44,692 | 95.9 | 1,890 | 4.1 |  |
|  | Outer regional | 26,925 | 95.7 | 1,213 | 4.3 |  | 27,905 | 95.8 | 1,234 | 4.2 |  | 27,367 | 95.2 | 1,384 | 4.8 |  |
|  | Remote | 5,654 | 95.9 | 242 | 4.1 |  | 5,841 | 95.5 | 273 | 4.5 |  | 5,768 | 95.7 | 259 | 4.3 |  |
|  | Very Remote | 4,119 | 95.7 | 185 | 4.3 |  | 4,372 | 95.7 | 199 | 4.4 |  | 3,915 | 94.8 | 215 | 5.2 |  |
|  |  |  |  |  |  |  |  |  |  |  |  |  |  |  |  |  |
| MACCS | 0 | 72,155 | 97.7 | 1,731 | 2.3 | <0.001 | 72,842 | 97.8 | 1,679 | 2.3 | <0.001 | 66,382 | 97.7 | 1,589 | 2.3 | <0.001 |
|  | 1-4 | 142,636 | 96.6 | 4,964 | 3.4 |  | 146,419 | 96.6 | 5,107 | 3.4 |  | 143,701 | 96.6 | 5,067 | 3.4 |  |
|  | 5-9 | 78,575 | 93.1 | 5,817 | 6.9 |  | 82,497 | 92.9 | 6,343 | 7.1 |  | 86,297 | 93.2 | 6,314 | 6.8 |  |
|  | 10+ | 15,151 | 85.1 | 2,653 | 14.9 |  | 16,411 | 84.5 | 3,006 | 15.5 |  | 18,016 | 84.5 | 3,318 | 15.6 |  |
|  |  |  |  |  |  |  |  |  |  |  |  |  |  |  |  |  |

**References**

1. Gray SL, Anderson ML, Dublin S, et al. Cumulative use of strong anticholinergics and incident dementia: a prospective cohort study. JAMA internal medicine. 2015;175:401-7; <https://doi.org/doi:https://dx.doi.org/10.1001/jamainternmed.2014.7663>.

2. Byrne CJ, Walsh C, Cahir C, et al. Anticholinergic and sedative drug burden in community-dwelling older people: a national database study. BMJ open. 2018;8:e022500; <https://doi.org/doi:https://dx.doi.org/10.1136/bmjopen-2018-022500>.

3. Ah Y-M, Suh Y, Jun K, et al. Effect of anticholinergic burden on treatment modification, delirium and mortality in newly diagnosed dementia patients starting a cholinesterase inhibitor: A population-based study. Basic & clinical pharmacology & toxicology. 2019;124:741-8; <https://doi.org/doi:https://dx.doi.org/10.1111/bcpt.13184>.

4. Boustani M, Campbell N, Munger S, et al. Impact of anticholinergics on the aging brain: A review and practical application. Aging Health. 2008;4:311-20; <https://doi.org/doi:https://dx.doi.org/10.2217/1745509X.4.3.311>.

5. Hwang S, Jun K, Ah Y-M, et al. Impact of anticholinergic burden on emergency department visits among older adults in Korea: A national population cohort study. Archives of Gerontology and Geriatrics. 2019;85:103912; <https://doi.org/https://doi.org/10.1016/j.archger.2019.103912>.

6. Rudolph JL, Salow MJ, Angelini MC, et al. The anticholinergic risk scale and anticholinergic adverse effects in older persons. Archives of internal medicine. 2008;168:508-13; <https://doi.org/doi:https://dx.doi.org/10.1001/archinternmed.2007.106>.

7. Coupland CAC, Hill T, Dening T, et al. Anticholinergic Drug Exposure and the Risk of Dementia: A Nested Case-Control Study. JAMA Internal Medicine. 2019;179:1084-93; <https://doi.org/doi:https://dx.doi.org/10.1001/jamainternmed.2019.0677>.

8. Hwang S, Chung JE, Jun K, et al. Comparative associations between anticholinergic burden and emergency department visits for anticholinergic adverse events in older Korean adults: a nested case-control study using national claims data for validation of a novel country-specific scale. BMC Pharmacology and Toxicology. 2021;22:2; <https://doi.org/10.1186/s40360-020-00467-6>.
